# *QuickStats*: Percentage* of Office-Based Primary Care Physicians Accepting New Patients, by Source of Payment Accepted — National Electronic Health Records Survey, 2015

**DOI:** 10.15585/mmwr.mm6628a9

**Published:** 2017-07-21

**Figure:**
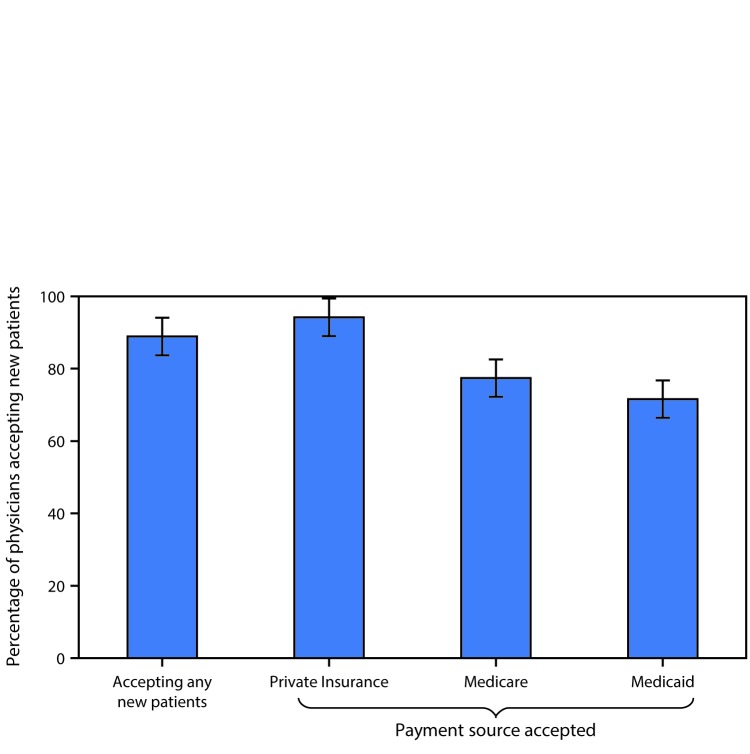
Overall, 88.9% of primary care physicians reported that they accepted new patients. However, acceptance varied by the patient’s expected payment source: 94.2% of physicians accepting new patients accepted privately insured patients, 77.4% accepted new Medicare patients, and 71.6% accepted new Medicaid patients. The percentages of primary care physicians accepting new Medicaid or Medicare patients were significantly lower than the percentage of primary care physicians accepting new privately insured patients.

Source: National Electronic Health Records Survey, 2015 data. Data available through the NCHS Research Data Center at https://www.cdc.gov/rdc/index.htm; survey questionnaire available at https://www.cdc.gov/nchs/ahcd/ahcd_questionnaires.htm.

